# Supplementary material for: Redox-mediated decoupled seawater direct splitting for H2 production
Source: Nat Commun. 2024 Oct 15;15:8874. doi: 10.1038/s41467-024-53335-w (PMC11473778; doi:10.1038/s41467-024-53335-w)
Supplement: Supplementary file 1 — Supplementary Information [file 41467_2024_53335_MOESM1_ESM.pdf]

# Supplementary Information

## Redox-Mediated Decoupled Seawater Direct Splitting for $H_2$ Production

Tao Liu[1,2,3,4✉]†, Cheng Lan[1,2,4✉]†, Min Tang[5]†, Mengxin Li[2,3], Yitao Xu[5], Hangrui Yang[6], Qingyue Deng[5], Wenchuan Jiang[1,2,4], Zhiyu Zhao[1,2,4], Yifan Wu[1,2,4✉], and Heping Xie[1,2,3,4,7✉]

1. State Key Laboratory of Intelligent Construction and Healthy Operation and Maintenance of Deep Underground Engineering, Sichuan University & Shenzhen University, Chengdu 610065, China.
2. Institute of New Energy and Low-Carbon Technology, Sichuan University, Chengdu 610065, China.
3. Guangdong Provincial Key Laboratory of Deep Earth Sciences and Geothermal Energy Exploitation and Utilization, Institute of Deep Earth Sciences and Green Energy, Shenzhen University, Shenzhen 518060, China.
4. Shenzhen Key Laboratory of Deep Engineering Science and Green Energy, Institute of Deep Earth Sciences and Green Energy, Shenzhen University, Shenzhen 518060, China.
5. Sichuan University-Pittsburgh Institute, Chengdu 610065, China.
6. School of Chemical Engineering, Sichuan University, Chengdu 610065, China.
7. College of Water Resource & Hydropower, Sichuan University, Chengdu 610065, China.

† These authors contributed equally to this work: Tao Liu, Cheng Lan, and Min Tang.

✉ Corresponding authors.

Correspondence should be addressed to: xiehp@scu.edu.cn, liutao3200023@scu.edu.cn, lancheng@scu.edu.cn, or fairwu@qq.com.

# Table of Contents


| | |
|---|---|
| Figure S1 | Page 3 |
| Figure S2 | Page 4 |
| Figure S3 | Page 5 |
| Figure S4 | Page 6 |
| Figure S5 | Page 7 |
| Figure S6 | Page 8 |
| Figure S7 | Page 9 |
| Figure S8 | Page 10 |
| Figure S9 | Page 11 |
| Figure S10 | Page 12 |
| Figure S11 | Page 13 |
| Figure S12 | Page 14 |
| Figure S13 | Page 15 |
| Figure S14 | Page 16 |
| Figure S15 | Page 17 |
| Figure S16 | Page 18 |
| Figure S17 | Page 19 |
| Figure S18 | Page 20 |
| Figure S19 | Page 21 |
| Figure S20 | Page 22 |
| Figure S21 | Page 23 |
| Figure S22 | Page 24 |
| Figure S23 | Page 25 |
| Figure S24 | Page 26 |
| Figure S25 | Page 27 |
| Figure S26 | Page 28 |
| Table S1 | Page 29 |
| Table S2 | Page 30 |

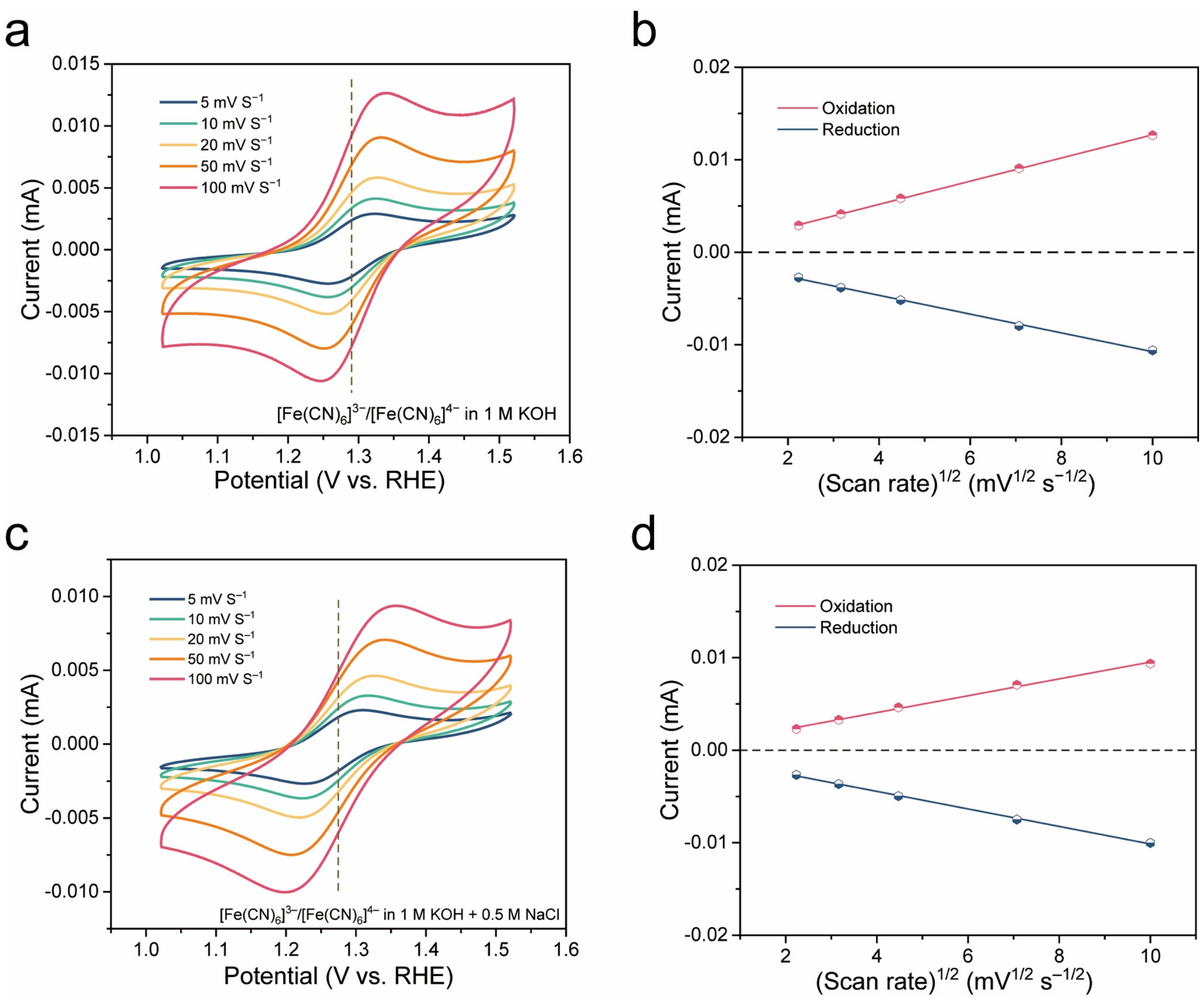


**Figure S1.** CV curves of $[Fe(CN)_6]^{3-/4-}$ at different scan rates in (a) 1 M KOH and (c) 1 M KOH + 0.5 M NaCl; the relationship between the oxidation peak current and reduction peak current with scan rates in (b) 1 M KOH and (d) 1 M KOH + 0.5 M NaCl. The experiments were carried out at room temperature (~25°C). And the working electrode was a glassy carbon electrode, with the carbon rod electrode serving as the counter electrode and the Ag/AgCl electrode (in saturated KCl solution) acting as the reference electrode.

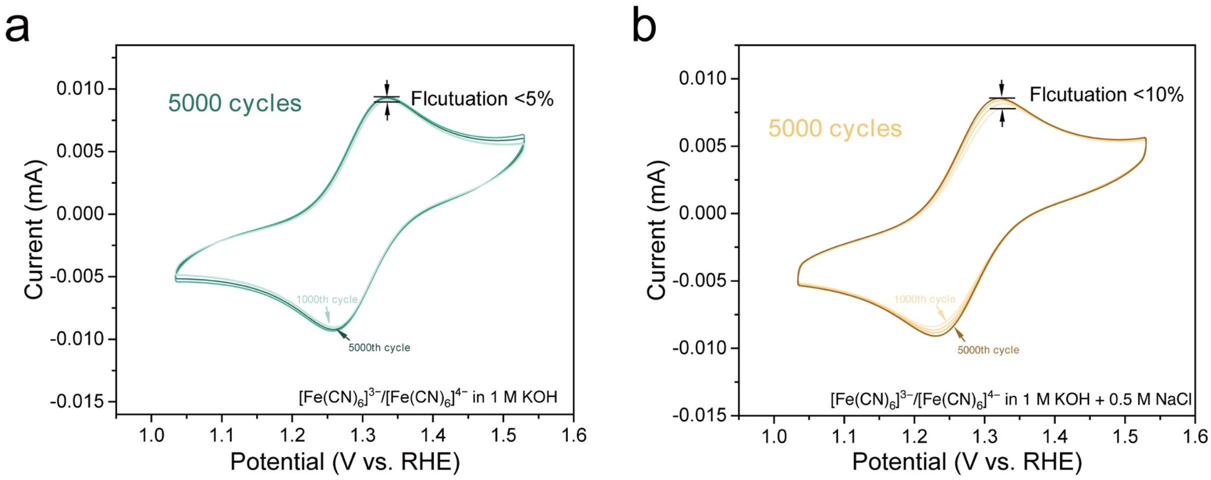


**Figure S2.** CV curves of 10 mM $[Fe(CN)_6]^{3-}$ in (a) 1 M KOH and (b) 1 M KOH + 0.5 M NaCl for 5000 cycles. The experiments were carried out at room temperature (~25°C). And the working electrode was a glassy carbon electrode, with the carbon rod electrode serving as the counter electrode and the Ag/AgCl electrode (in saturated KCl solution) acting as the reference electrode.

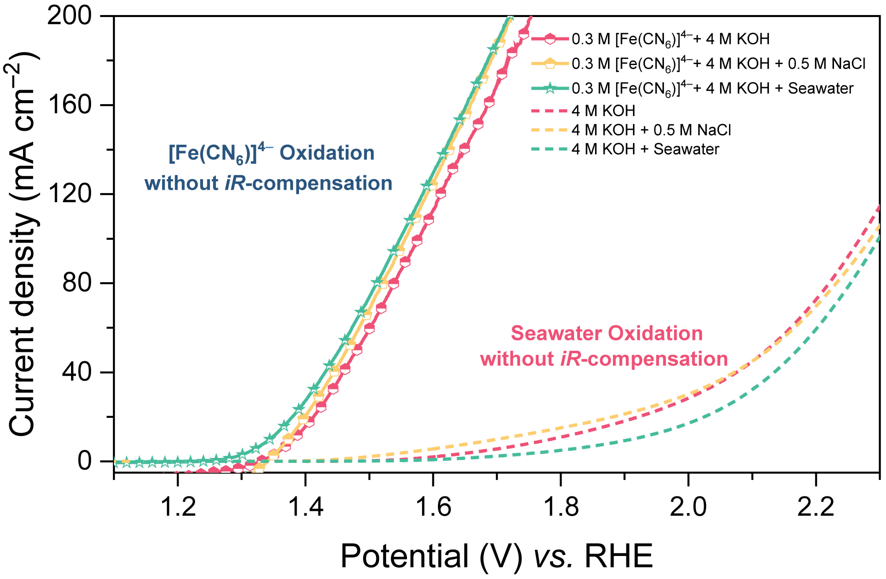


**Figure S3.** LSV curves of the carbon felt electrode for $[Fe(CN)_6]^{4-}$ oxidation and seawater oxidation in different electrolytes without *iR* compensation at a scan rate of 2 mV $s^{-1}$. The carbon rod electrode acted as the counter electrode, and the Hg/HgO electrode served as the reference electrode at 25°C.

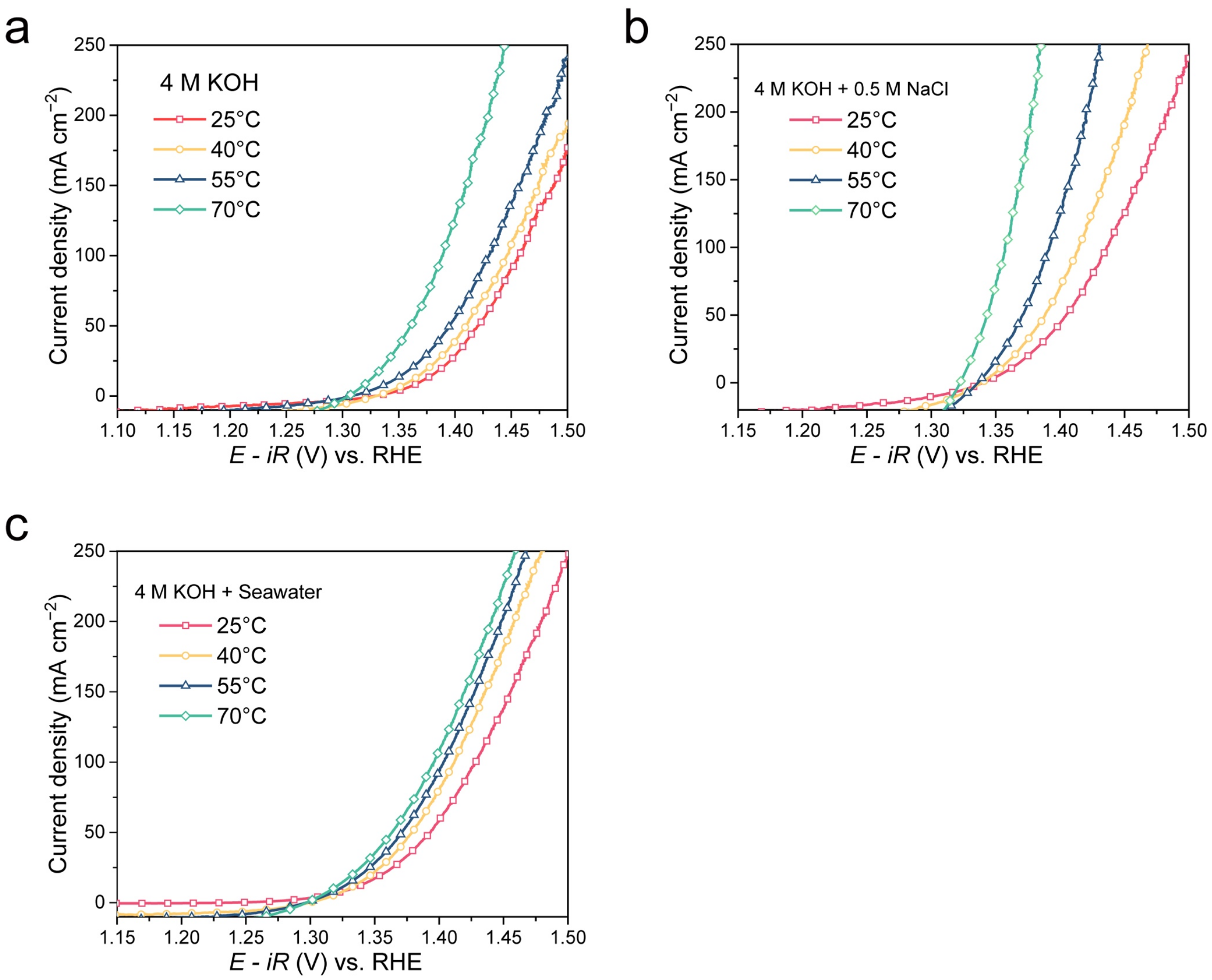


**Figure S4.** LSV curves of the carbon felt electrode for $[Fe(CN)_6]^{4-}$ oxidation with 100% iR compensation at different temperatures in (a) 4 M KOH, (b) 4 M KOH + 0.5 M NaCl, and (c) 4 M KOH + seawater, where $R$ was determined to be 1.2 ± 0.1 Ω. The carbon rod electrode acted as the counter electrode, and the Hg/HgO electrode served as the reference electrode.

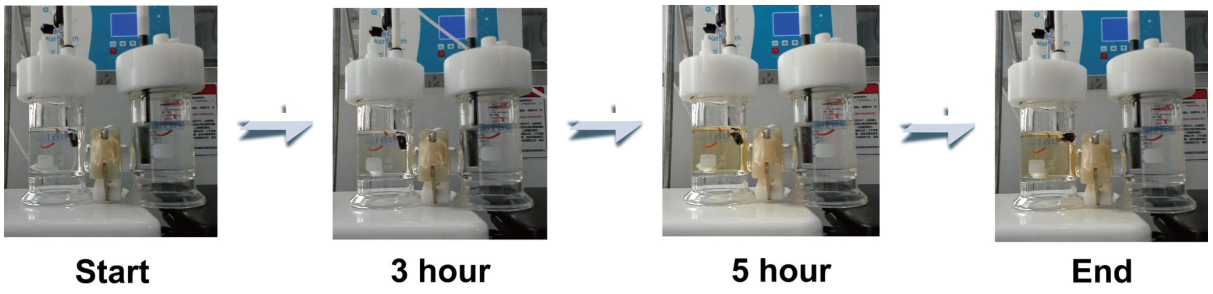


**Figure S5.** Photos of the carbon felt electrode at various times during electrolysis.

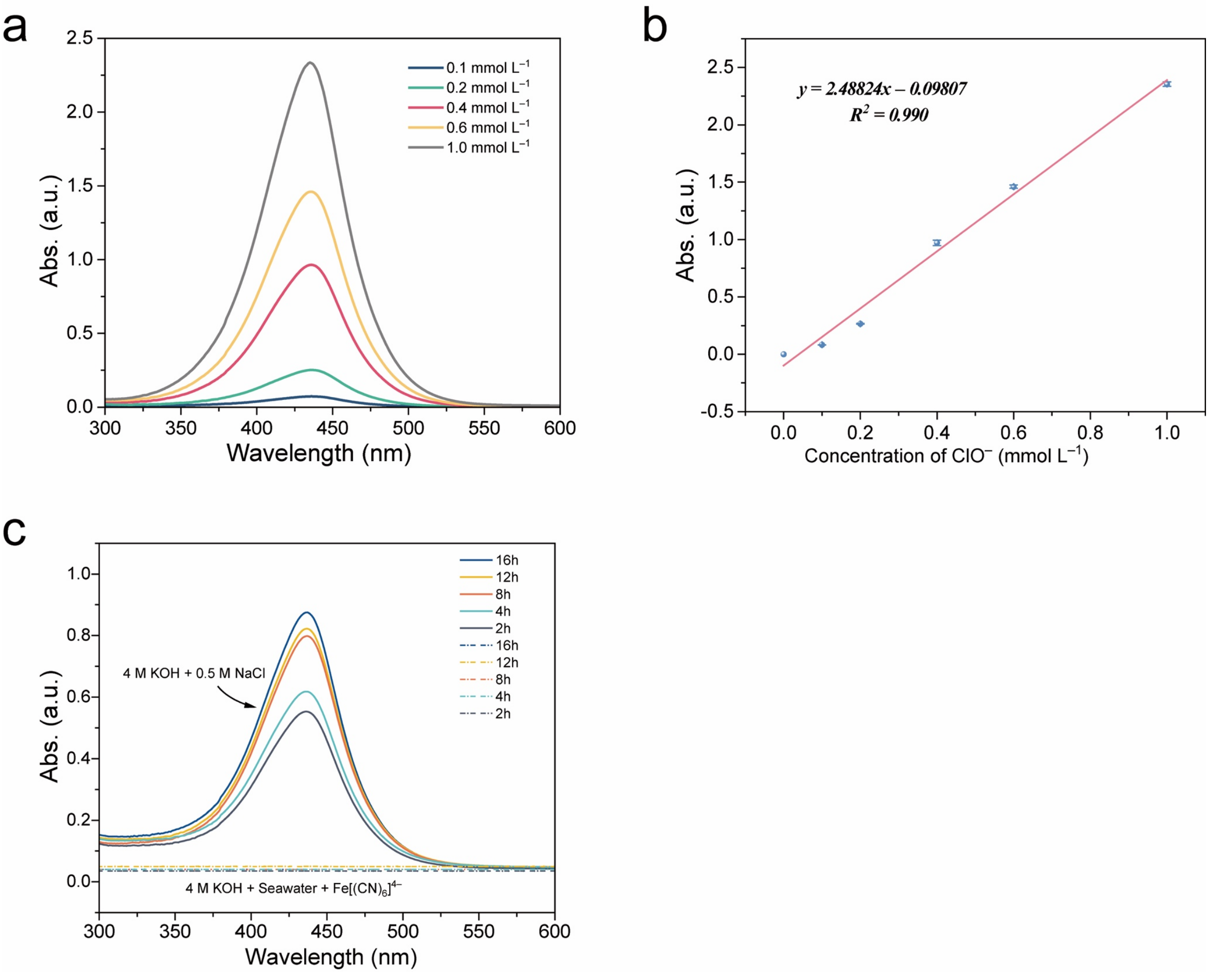


**Figure S6.** (a) UV–vis absorption spectra of the solutions containing 0.5 mL of o-tolidine in DI water (10 mL) with different hypochlorite concentrations; these concentrations are obtained by collecting different amounts of $ClO^-$ (0.1, 0.2, 0.4, 0.6, and 1.0 mmol $L^{-1}$) in 4 M KOH. (b) Calibration curve obtained by plotting the hypochlorite concentration against the absorption peak intensity at $\lambda = 436$ nm. (c) UV–vis absorption spectra of the $ClO^-$ concentration change in the anolyte from 0.3 M $[Fe(CN)_6]^{4-}$ + 4 M KOH + seawater and 4 M KOH + 0.5 M NaCl.

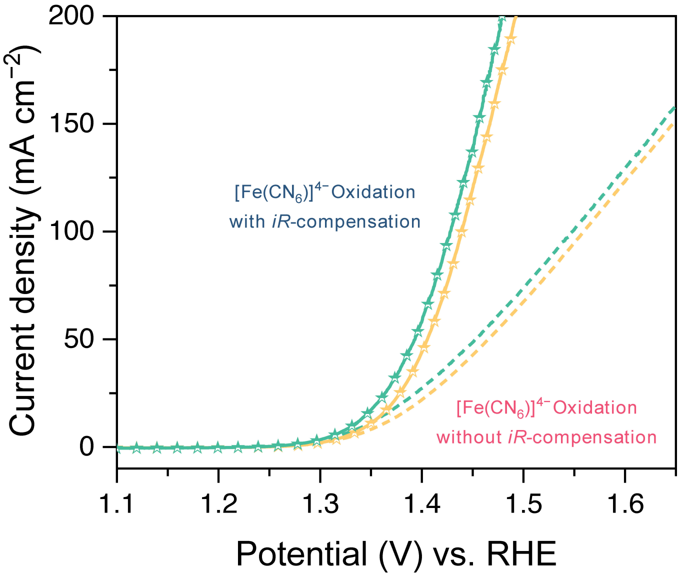


**Figure S7.** LSV curves of the carbon felt electrode for $[Fe(CN)_6]^{4-}$ oxidation with/without 100% *iR* compensation in 4 M KOH + seawater and 4 M KOH + $Cl^-$-saturated seawater at a scan rate of 2 mV $s^{-1}$, where *R* was determined to be 1.2 ± 0.1 Ω. The carbon rod electrode acted as the counter electrode, and the Hg/HgO electrode served as the reference electrode at 25°C.

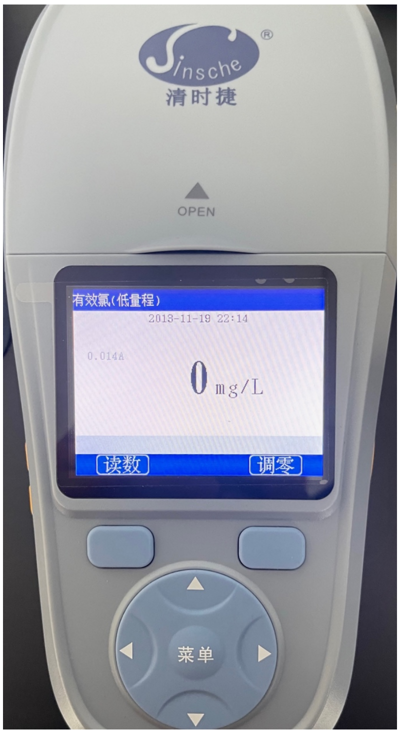


**Figure S8.** $ClO^-$ concentration tests. The detector displays the concentration of $ClO^-$ (0 mg $L^{-1}$). The color of the electrolyte remained unaltered after adding a commercial color-indicating solution and a commercial pH-adjusting solution.

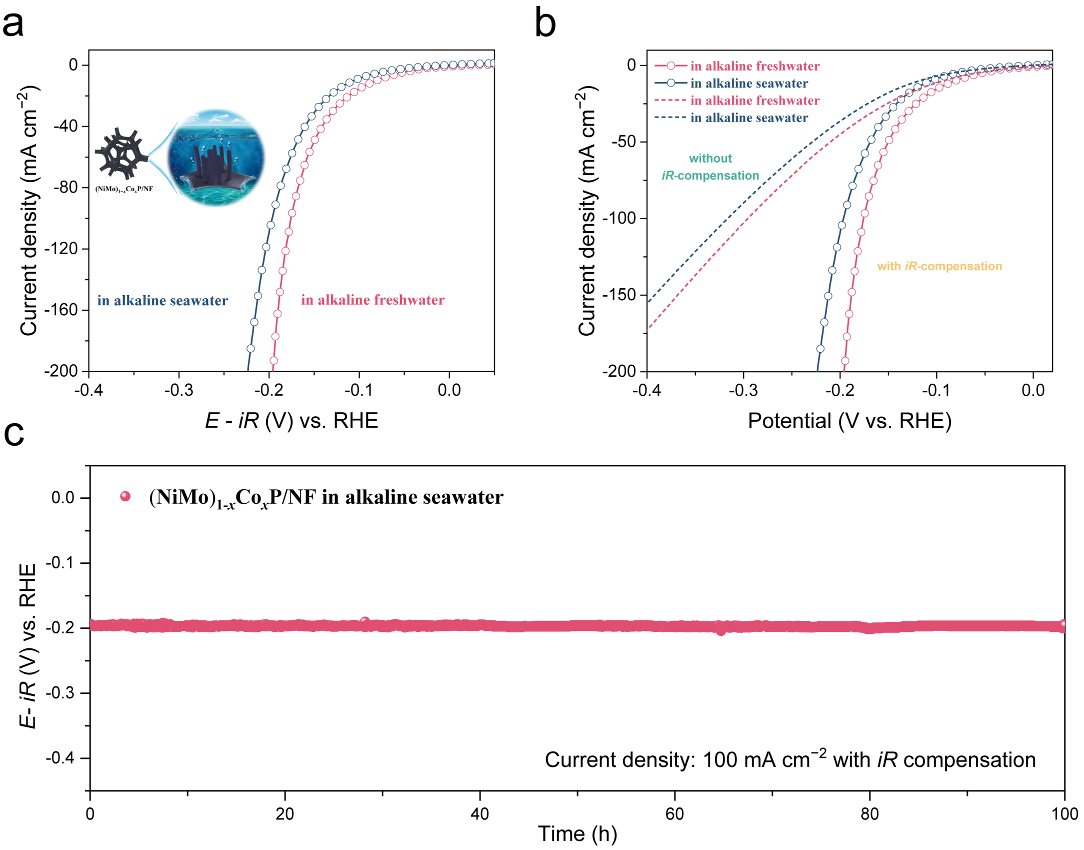


**Figure S9.** (a) Polarization curves of $(NiMo)_{1-x}Co_xP$/NF for the HER in alkaline freshwater and alkaline seawater with 100% *iR* compensation, where *R* was determined to be 1.2 ± 0.1 Ω.; (b) Polarization curves of $(NiMo)_{1-x}Co_xP$/NF for the HER with/without *iR* correction in alkaline freshwater and alkaline seawater (c) HER chronopotentiometry curve of $(NiMo)_{1-x}Co_xP$/NF recorded at a current density of 100 mA cm$^{-2}$ in alkaline seawater. The experiments were carried out at room temperature (~25°C). And the carbon rod electrode acted as the counter electrode, and the Hg/HgO electrode served as the reference electrode.

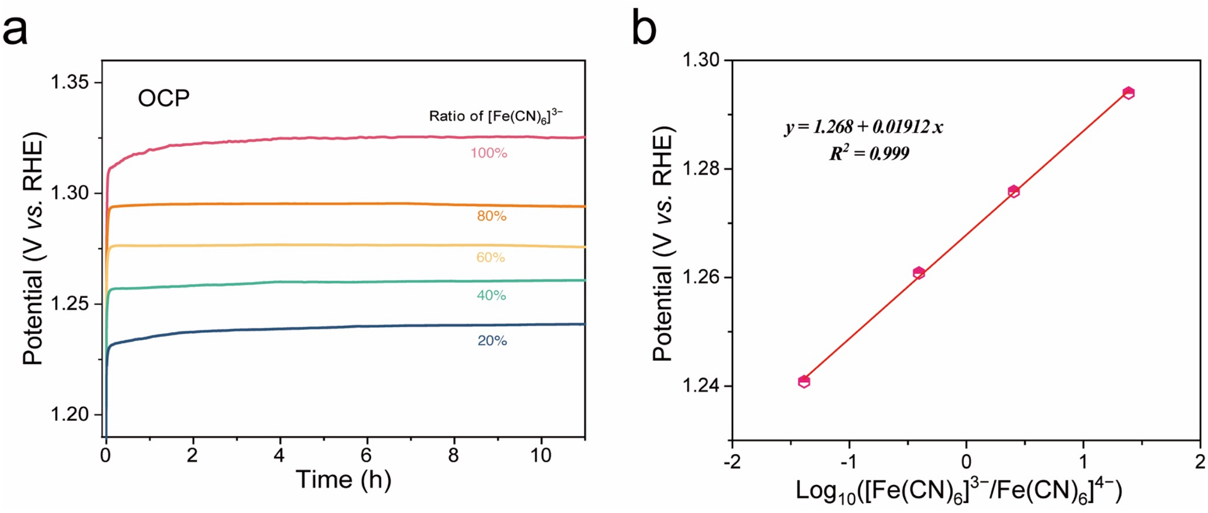


**Figure S10.** (a) Open circuit potential curves of $[Fe(CN)_6]^{3-/4-}$ in alkaline seawater solutions; (b) Calibration curves for potentials of $[Fe(CN)_6]^{3-/4-}$ in alkaline seawater solution with respect to the relative amounts of the $[Fe(CN)_6]^{3-}$and $[Fe(CN)_6]^{4-}$ species. The experiments were carried out at room temperature (~25°C). And the carbon rod electrode acted as the counter electrode, and the Hg/HgO electrode served as the reference electrode.

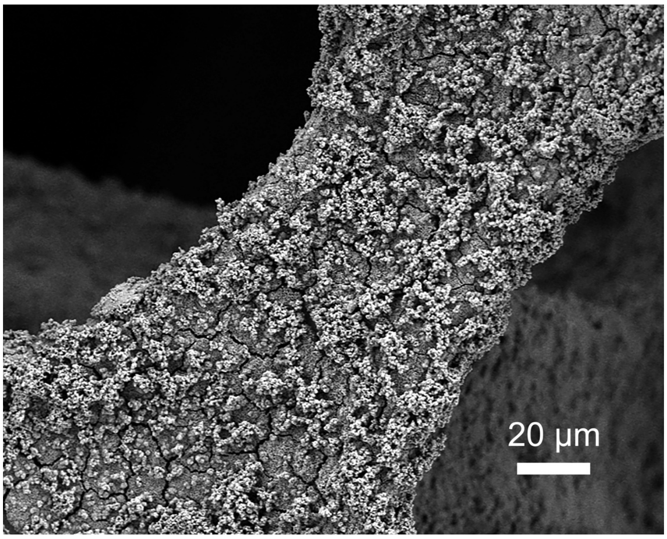


**Figure S11.** Low-resolution SEM image of Fe-$Ni(OH)_2$/NF.

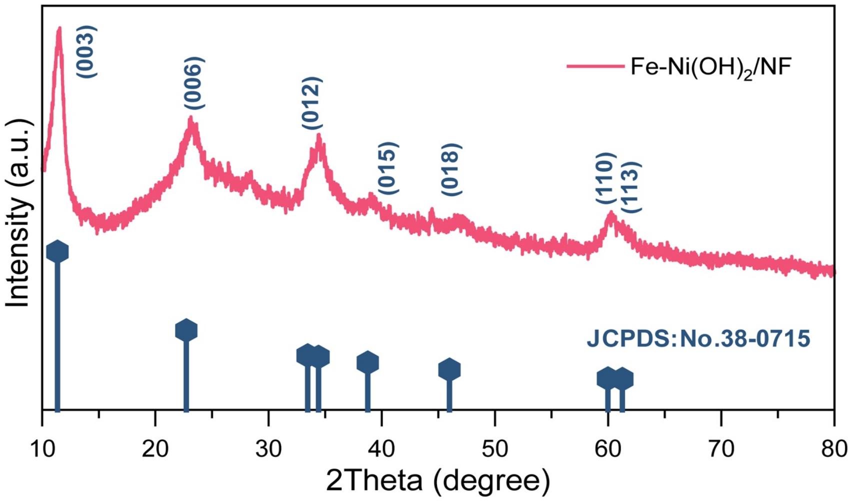


**Figure S12.** XRD pattern of Fe-$Ni(OH)_2$/NF.

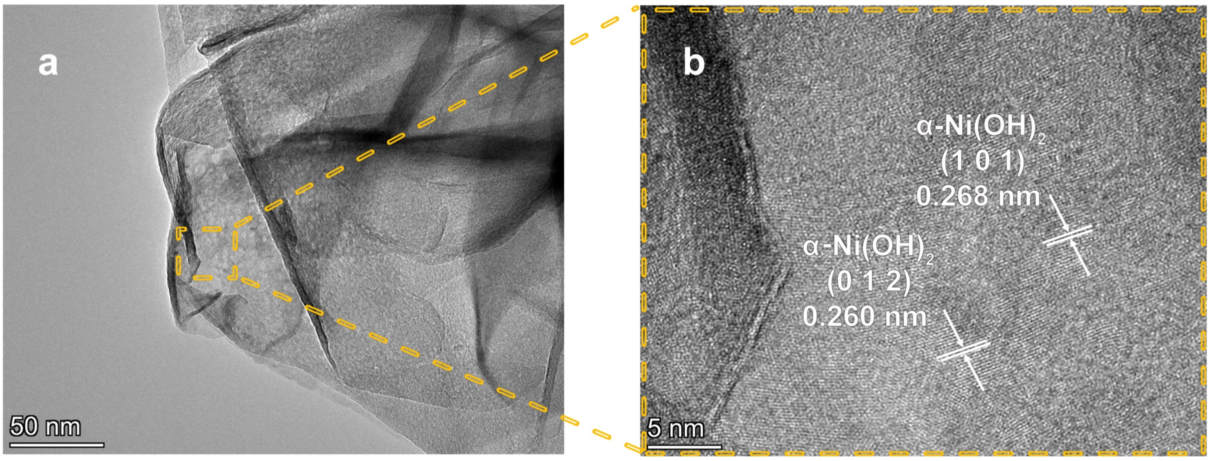


**Figure S13.** (a) TEM image and (b) high-resolution TEM image of Fe-Ni$(OH)_2$/NF.

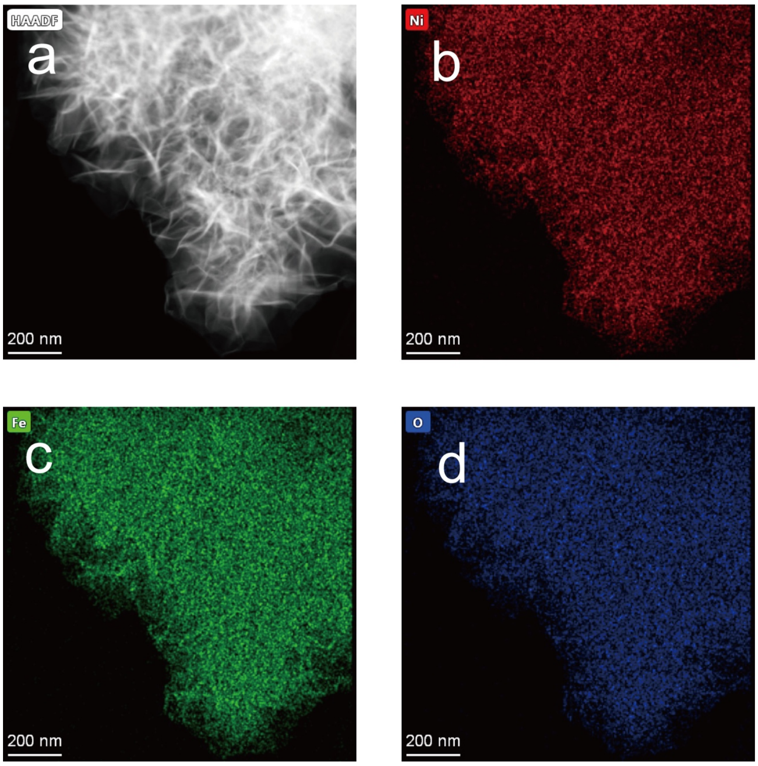


**Figure S14.** (a) HAADF-STEM image of Fe-Ni$(OH)_2$/NF. (b-d) Corresponding EDX elemental maps of the Ni, Fe, and O contents.

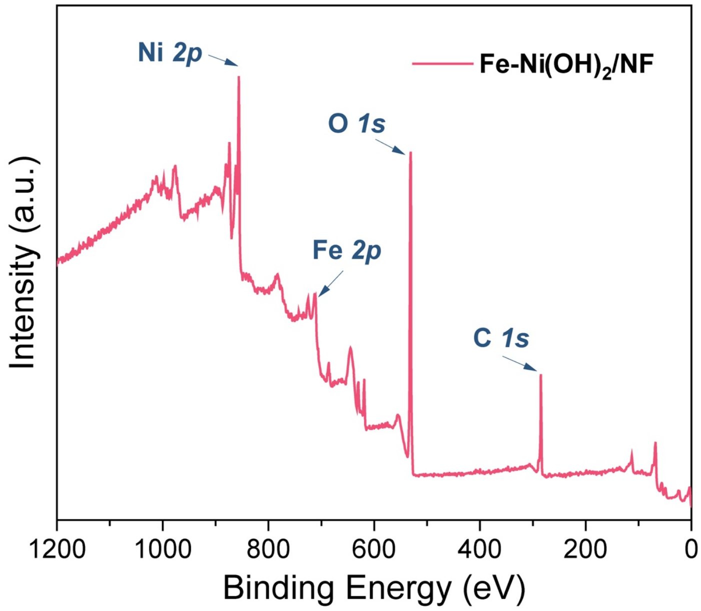


**Figure S15.** XPS survey spectrum of $Fe\text{-}Ni(OH)_2/NF$.

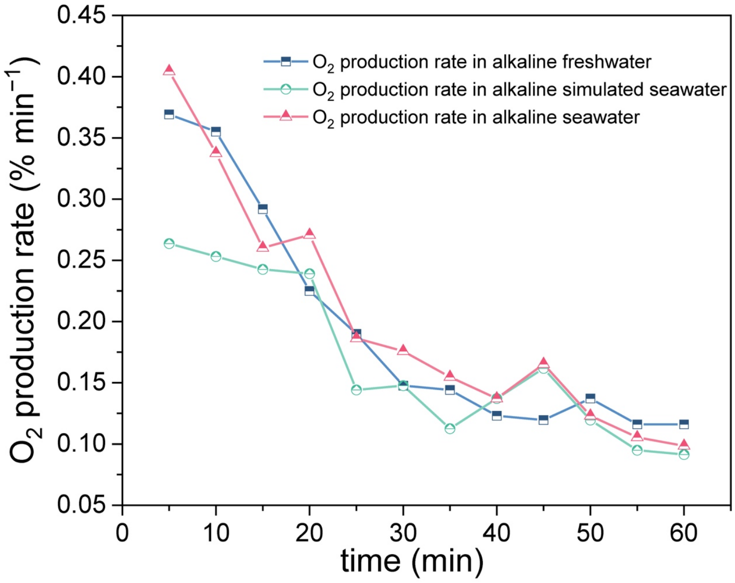


**Figure S16.** $O_2$ production rates over time in different solutions.

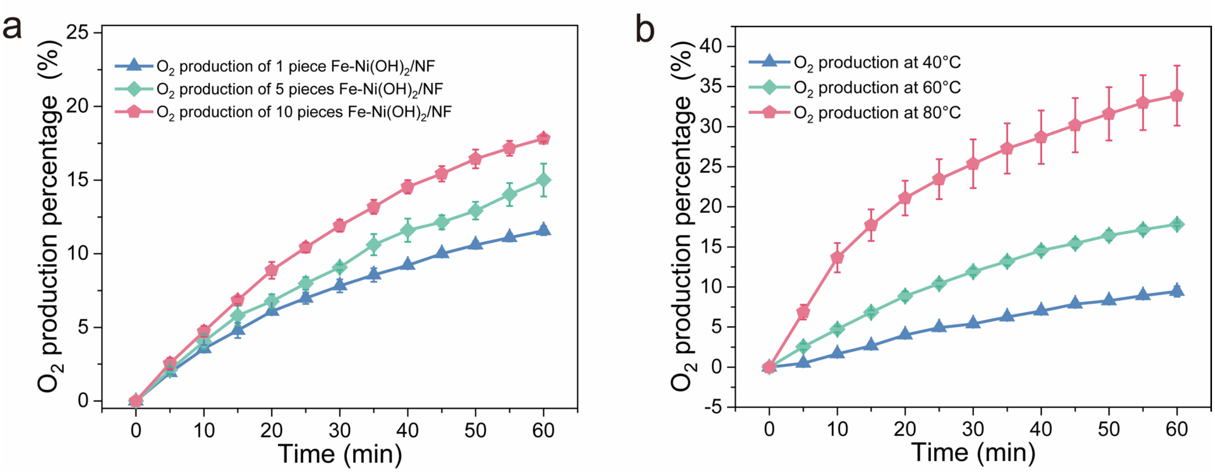


**Figure S17.** Variation in the $O_2$ production over time using (a) different amounts of catalyst and (b) different temperatures (pi error bar).

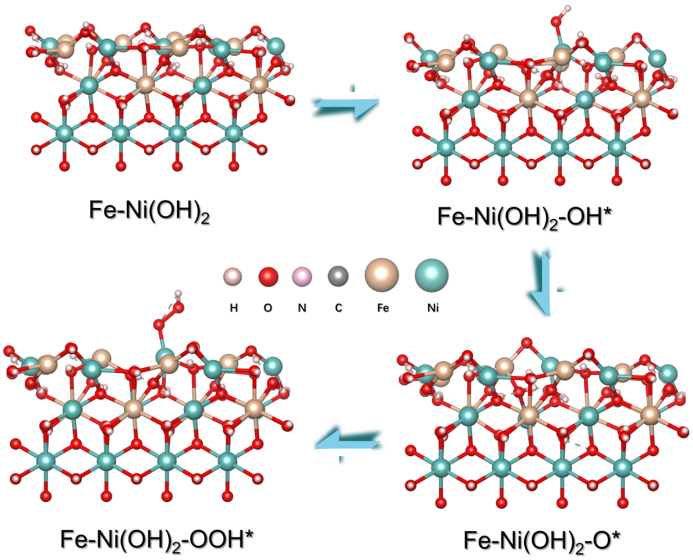


**Figure S18.** OER pathways on the Fe-$Ni(OH)_2$/NF catalyst without $[Fe(CN)_6]^{3-}$. The silver, red, pink, grey, brown, and green balls represent the H, O, N, C, Fe, and Ni atoms, respectively.

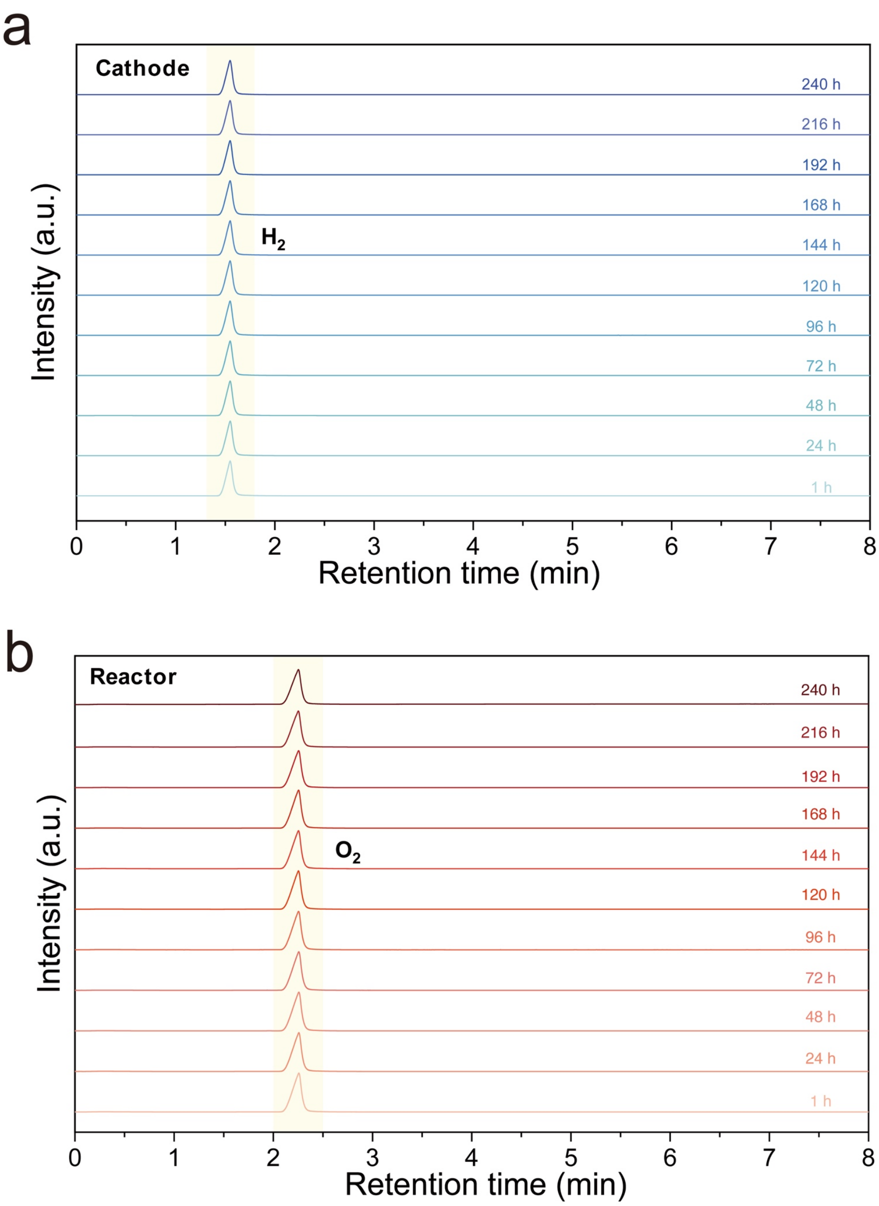


**Figure S19.** Original chromatograms for (a) hydrogen and (b) oxygen evolution during decoupled seawater direct electrolysis.

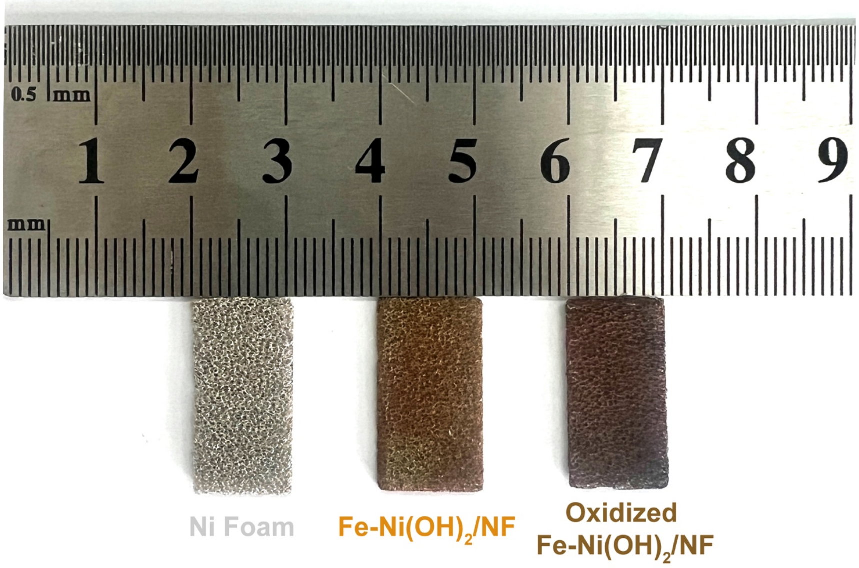


**Figure S20.** Photographs of the Ni foam, Fe-$Ni(OH)_2$/NF and oxidized Fe-$Ni(OH)_2$/NF.

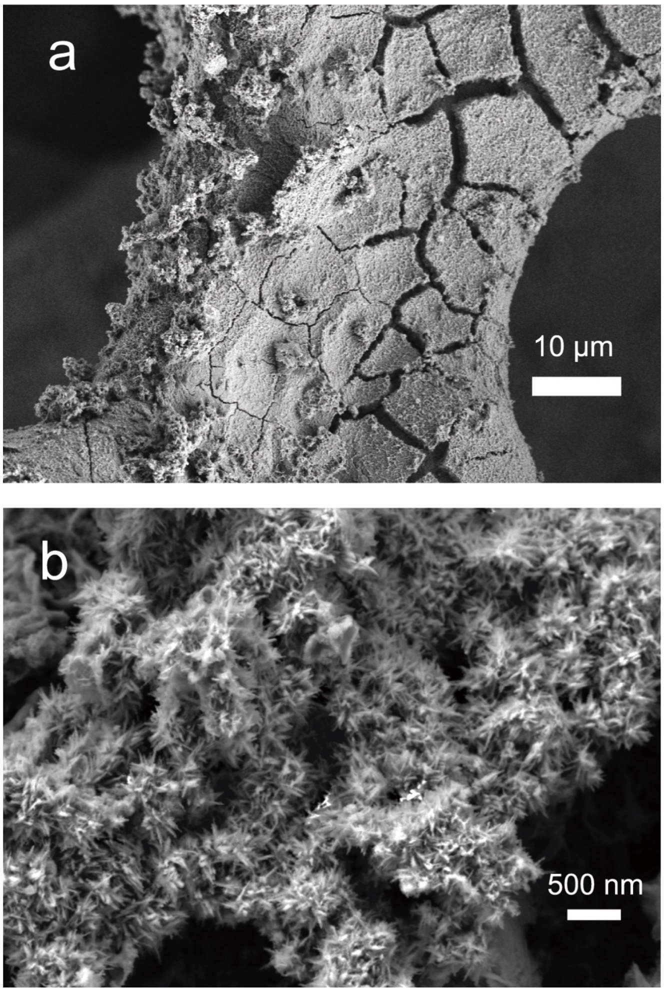


**Figure S21.** (a) Low-resolution and (b) high-resolution SEM images of Fe-Ni$(OH)_2$/NF after long-term reaction.

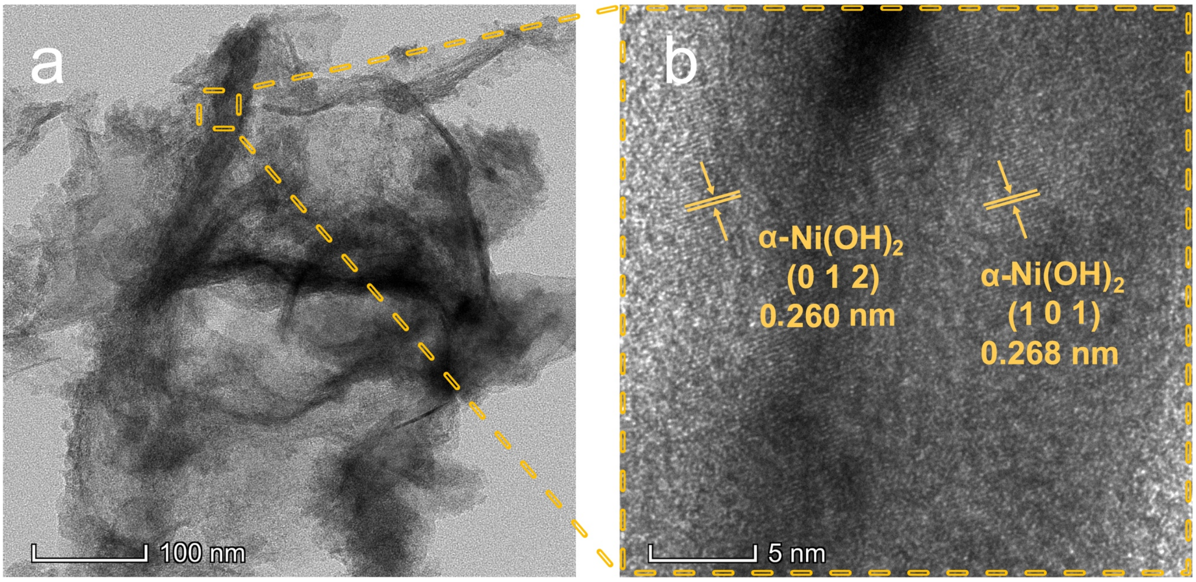


**Figure S22.** (a) TEM image and (b) high-resolution TEM image of Fe-$Ni(OH)_2$/NF after the reduction reaction.

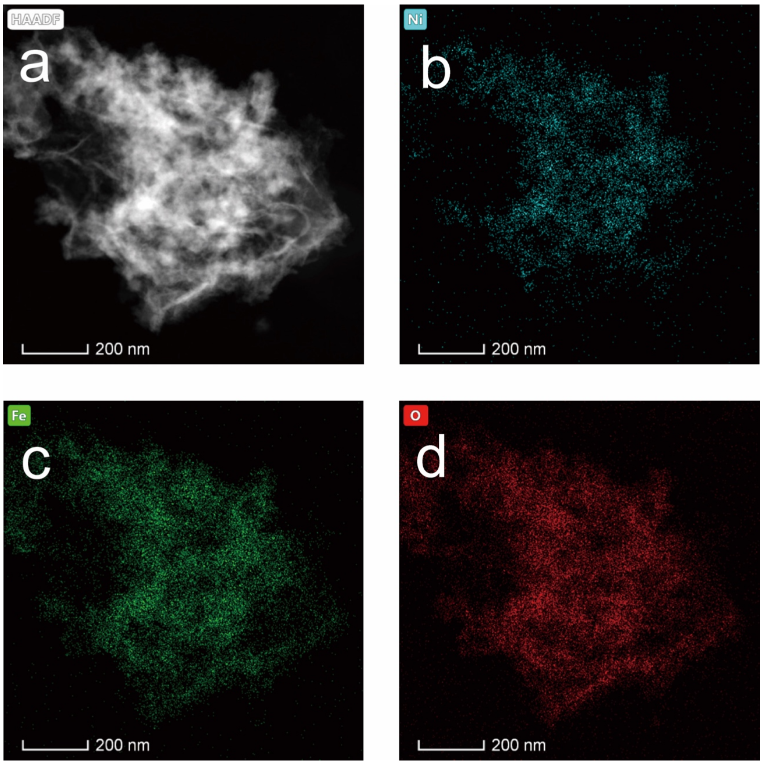


**Figure S23.** (a) HAADF-STEM image of Fe-$Ni(OH)_2$/NF after the reduction reaction. (b-d) Corresponding EDX elemental maps of the Ni, Fe, and O contents.

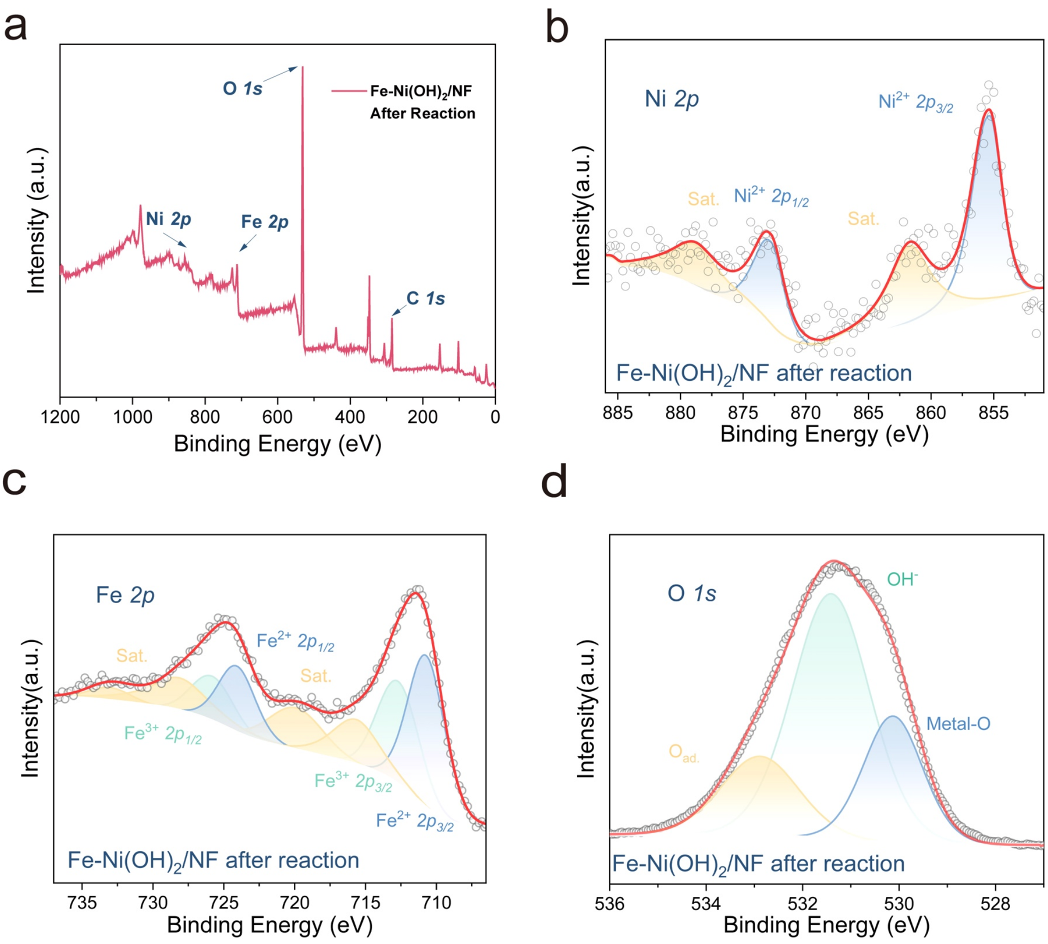


**Figure S24.** (a) XPS survey spectrum of Fe-Ni$(OH)_2$/NF after long-term reaction and (b) Ni *2p*, (c) Fe *2p*, and (d) O *1s* XPS spectra of Fe-Ni$(OH)_2$/NF after long-term reaction.

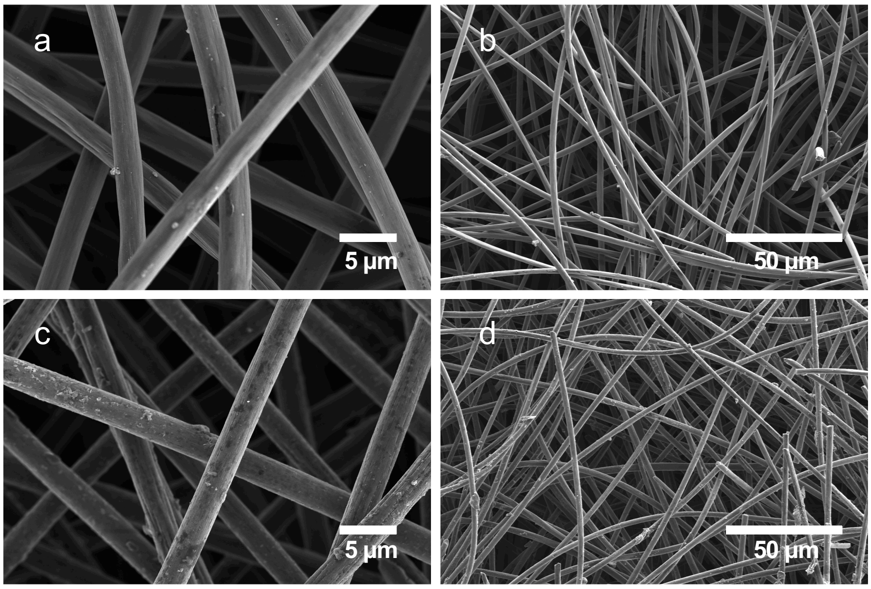


**Figure S25.** (a) High-resolution and (b) low-resolution SEM images of the carbon felt electrode before the long-term electrolysis in $Cl^-$-saturated alkaline seawater. (c) High-resolution and (d) low-resolution SEM images of the carbon felt electrode after long-term electrolysis in $Cl^-$-saturated alkaline seawater.

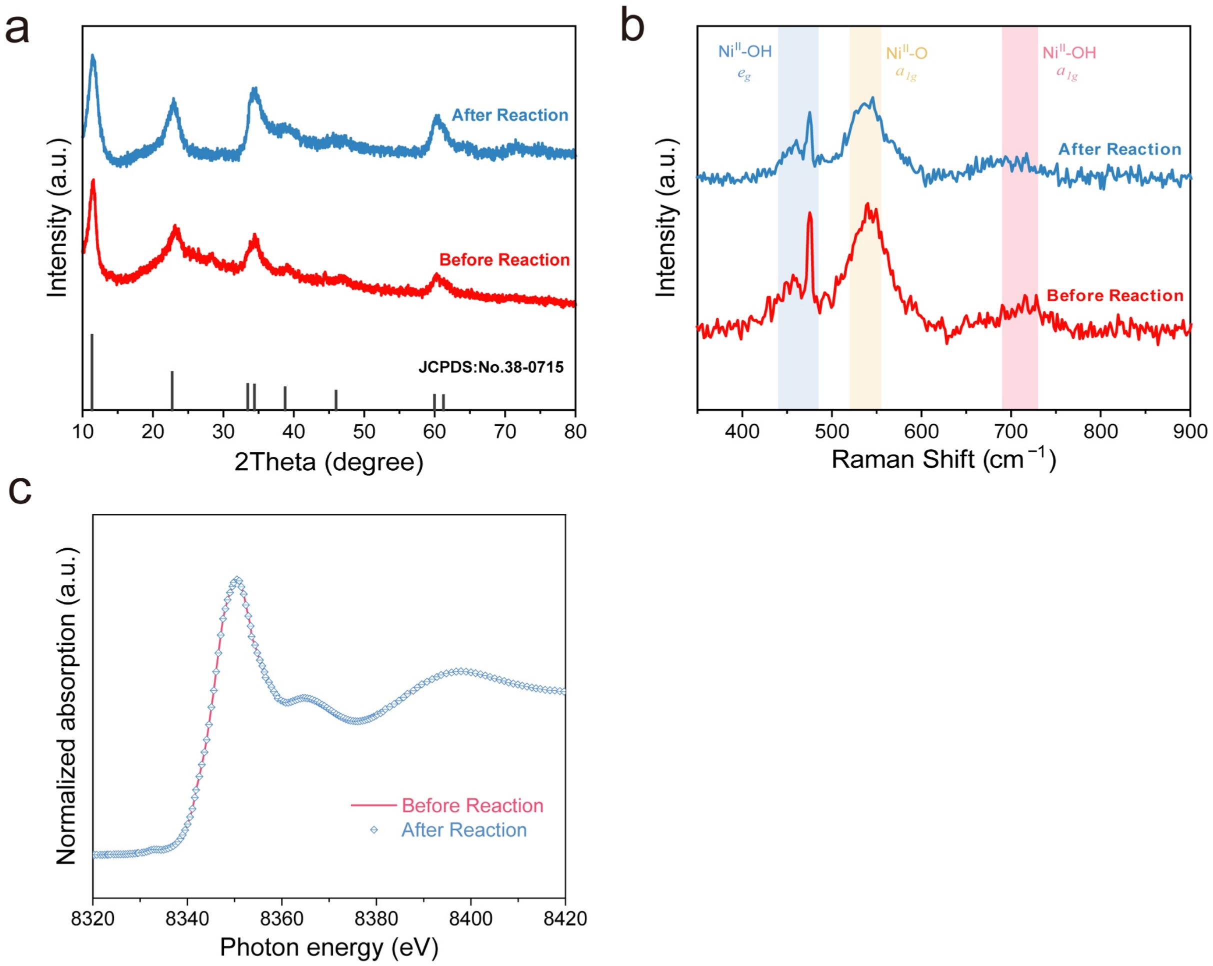


**Figure S26.** (a) XRD pattern, (b) Raman spectra, and (c) Ni K-edge XANES spectra of Fe-$Ni(OH)_2$/NF before and after reaction.

**Table S1.** The composition of seawater from Shenzhen Bay, China. Data were sourced from our previous work: *Nature 2022, 612, 673-678*.

| ***Species*** | ***Conc.[mg/L]*** |
|---|---|
| **$Cl^{-}$** | 10108 |
| **$Na^{+}$** | 5648 |
| **$SO_4^{2-}$** | 1982 |
| **$Mg^{2+}$** | 690 |
| **$Ca^{2+}$** | 218 |
| **$K^{+}$** | 244 |
| **$Br^{-}$** | 26.45 |
| **$Sr^{2+}$** | 3.77 |
| **$F^{-}$** | 1.7 |
| **Total dissolved solids** | 21028 |
| **Organic matter** | 11.6 |
| **Microbe** | 6 (CFU/mL) |

**Table S2.** A comparison of the DSDE system with the state-of-the-art seawater electrolyzer in the cell voltage and electricity expense.

| *Electrolyzer* | *Electrolyte* | *Current density (mA cm$^{-2}$)* | *Cell Voltage (V)* | *Basic electricity expense (kWh m$^{-3}$ $H_2$)* | *Reference* |
|---|---|---|---|---|---|
| **NiFe-NiS*x*/NF (+) \|\| Ni-NiO-$Cr_2O_3$/NF (–)** | 1 M KOH + 0.5 M NaCl | 400 | 2.12 | 5.07 | *Natl. Acad. Sci. 2019, 116, 6624-6629* |
| **S-(Ni,Fe)OOH/NF (+, –)** | 1 M KOH + Seawater | 500 | 1.837 | 4.40 | *Energy Environ. Sci. 2020,13, 3439-3446* |
| **BSCF@$CeO_2$@NiFe (+) \|\| Pt/C@NF (–)** | 1 M KOH + Seawater | 100 | 1.76 | 4.21 | *Processes. 2022, 10, 668* |
| **Se-FeCo-LDH (+, –)** | 1 M KOH + Seawater | 20 | 1.646 | 3.94 | *Journal of Colloid and Interface Science. 2023, 650, 0021-9797* |
| **S,P-(Ni,Mo,Fe)OOH/ NiMoP/wood aerogel (+, –)** | 1 M KOH + Seawater | 500 | 1.861 | 4.45 | *Environmental. 2021, 293, 120215* |
| **NRAHM-NiO (+, –)** | 1 M KOH + 0.5 M NaCl | 500 | 2.01 | 4.81 | *ACS Catal. 2023, 13, 8* |
| **$Ni_3S_2$/$Co_3S_4$ (+, –)** | 1 M KOH + Seawater | 800 | 2.08 | 4.98 | *Environmental. 2021, 291, 120071* |
| **Ir/C/NF (+) \|\| MoNi/$NiMoO_4$ (–)** | 1 M KOH + 0.5 M NaCl | 10 | 2.1 | 5.03 | *Nano Energy, 2022, 98,107295* |
| **NiFe-LDH (+) \|\| Pt/C (–)** | 0.5 M KOH (+) \|\| 0.5 M NaCl (–) | 275 | 1.7 | 4.07 | *Energy Environ. Sci. 2020,13, 1725* |
| **NiMoN@NiFeN/NF (+) \|\| NiMoN/NF (–)** | 1 M KOH + seawater | 500 | 1.8 | 4.31 | *Nat. Commun. 2019, 10, 5106* |
| **NiFe LDH (+) \|\| Pt/C (–)** | 1 M KOH + 0.5 M NaCl | 200 | 1.6 | 3.83 | *Adv. Energy Mater. 2018, 8, 1800338* |

| | | | | | |
|---|---|---|---|---|---|
| **Co-Se1(+) \|\| Co-Se4 (–)** | Seawater | 20 | 2 | 4.79 | *Adv. Energy Mater. 2018, 8, 1801926* |
| **NiNS/NF (+, –)** | Seawater | 70 | 2 | 4.79 | *J. Mater. Chem. A 2019, 7, 811* |
| **S-(Ni,Fe)OOH/NF (+) \|\|NiCoN\|Ni*x*P\|NiCoN/ NF (–)** | Seawater | 10 | 1.81 | 4.33 | *ACS Energy Lett. 2020, 5, 2681* |
| **Fe-$Co_2P$ (+, –)** | Seawater | 10 | 1.72 | 4.12 | *J. Mater. Chem. A. 2021, 9, 1418-1428* |
| **Solid hygroscopic SDE** | Seawater | 250 | 1.85 | 4.43 | *Nature 2022, 612, 673-678* |
| **Pt/C/NF (+) \|\| Ce-NiFe LDH/NF (–)** | 1 M KOH + seawater | 200 | 2.07 | 4.95 | *J. Ener. Chem. 2024, 91, 306-312* |
| **Pt/C/NF (+) \|\| Ce-NiFe LDH/NF (–)** | 1 M KOH + seawater | 300 | 2.24 | 5.36 | *J. Ener. Chem. 2024, 91, 306-312* |
| **Pt/C/NF (+) \|\| (NiFe)$C_2O_4$/NF (–)** | 1 M KOH + seawater | 100 | 1.8 | 4.31 | *Angew. 2024, 63, e202316522* |
| **Pt/C/NF (+) \|\| (NiFe)$C_2O_4$/NF (–)** | 1 M KOH + seawater | 300 | 2.1 | 5.03 | *Angew. 2024, 63, e202316523* |
| **Pt/C/NF (+) \|\| (NiFe)$C_2O_4$/NF (–)** | 1 M KOH + seawater | 500 | 2.4 | 5.74 | *Angew. 2024, 63, e202316524* |
| **DSDE system** | 4 M KOH + seawater | 10 | 1.37 | 3.28 | *This Work* |
| **DSDE system** | 4 M KOH + seawater | 100 | 1.57 | 3.76 | *This Work* |
| **DSDE system** | 4 M KOH + seawater | 200 | 1.65 | 3.95 | *This Work* |
| **DSDE system** | 4 M KOH + seawater | 400 | 1.82 | 4.36 | *This Work* |
