# Supplementary material for: Redox-mediated decoupled seawater direct splitting for H2 production
Source: Nat Commun. 2024 Oct 15;15:8874. doi: 10.1038/s41467-024-53335-w (PMC11473778; doi:10.1038/s41467-024-53335-w)
Supplement: Supplementary file 3 — Description of Additional Supplementary Files [file 41467_2024_53335_MOESM3_ESM.pdf]

Description of Additional Supplementary Files

File Name: Supplementary Data 1

- Fe-CONTCAR;

  Description: $[Fe(CN)_6]^{3-}$ loaded on the Fe-$Ni(OH)_2$/NF structure-surface model.

- Fe-O-CONTCAR;

  Description: $[Fe(CN)_6]^{3-}$ loaded on the Fe-$Ni(OH)_2$/NF structure with adsorbed O* surface model.

- Fe-O-O-H- CONTCAR;

  Description: $[Fe(CN)_6]^{3-}$ loaded on the Fe-$Ni(OH)_2$/NF structure with adsorbed OOH* surface model.

- Fe-OH-CONTCAR;

  Description: $[Fe(CN)_6]^{3-}$ loaded on the Fe-$Ni(OH)_2$/NF structure with adsorbed OH* surface model.

- O-CONTCAR;

  Description: Fe-$Ni(OH)_2$/NF structure with adsorbed O* surface model.

- OH-CONTCAR;

  Description: Fe-$Ni(OH)_2$/NF structure with adsorbed OH* surface model.

- OOH-CONTCAR;

  Description: Fe-$Ni(OH)_2$/NF structure with adsorbed OOH* surface model.

- slab-CONTCAR;

  Description: Fe-$Ni(OH)_2$/NF structure-surface model.

File Name: Supplementary Movie 1. O_2 gas evolution;

Description: O_2 gas evolution movie of the DSDE system.
